# External Mechanical Microstimuli Modulate the Osseointegration of Titanium Implants in Rat Tibiae

**DOI:** 10.1155/2013/234093

**Published:** 2013-12-03

**Authors:** Giovanna Zacchetti, Anselm Wiskott, Joël Cugnoni, John Botsis, Patrick Ammann

**Affiliations:** ^1^Division of Bone Diseases, Department of Internal Medicine and Faculty of Medicine, University Hospital of Geneva, 1211 Geneva, Switzerland; ^2^Laboratory of Biomaterials, School of Dentistry, University of Geneva, 1205 Geneva, Switzerland; ^3^Laboratory of Applied Mechanics and Reliability Analysis, École Polytechnique Fédérale de Lausanne, 1015 Lausanne, Switzerland; ^4^Division of Bone Diseases, Department of Rehabilitation and Geriatrics, Av de la Roseraie 64, University Hospital of Geneva, 1211 Geneva, Switzerland

## Abstract

*Purpose*. To assess the effect of external mechanical microstimuli of controlled magnitude on the microarchitecture of the peri-implant bone beds in rat tibiae. *Materials and Methods*. Tibiae of forty rats were fitted with two transcutaneous titanium cylinders. After healing, the implants were loaded to 1 to 3 N, five days/week for four weeks. These force levels translated into intraosseous strains of 700 ± 200 *με*, 1400 ± 400 *με*, and 2100 ± 600 *με*. After sacrifice, the implants' pullout strength was assessed. Second, the bone's microarchitecture was analyzed by microcomputed tomography (*μ*CT) in three discrete regions of interest (ROIs). Third, the effect of loading on bone material properties was determined by nanoindentation. *Results*. The trabecular BV/TV significantly increased in an ROI of 0.98 mm away from the test implant in the 1 N versus the 3 N group with an opposite trend for cortical thickness. Pull-out strength significantly increased in the 2 N relatively to the nonstimulated group. Higher values of E-modulus and hardness were observed in the trabecular bone of the 2 N group. *Conclusion*. The *in vivo* mechanical loading of implants induces load-dependent modifications in bone microarchitecture and bone material properties in rat tibiae. In pull-out strength measurements, implant osseointegration was maximized at 2 N (1400 ± 400 *με*).

## 1. Introduction

The endosseous insertion of titanium implants is common practice in dentistry and orthopedics. After the placement procedure, optimal osseointegration is crucial for the mechanical stability of the implant in the bone bed. Yet this process is greatly influenced by the factors affecting the interplay between the implant and the surrounding bone. In particular, diseases such as osteoporosis or an insufficient dietary protein intake negatively impact bone quality and impair implant osseointegration [1]. Conversely, in a rat model, the systemic treatment with antiosteoporotic agents improves implant osseointegration as evidenced by the increase in pull-out strength due to a favorable effect on microarchitecture and the intrinsic quality of the bone tissue that develops around the implants [2, 3].

The mechanical loading of bone *in vivo* positively affects bone mass as it enhances bone formation relatively to bone resorption. Further, it translates into an increased BMD and an improved trabecular and cortical microarchitecture—all parameters which jointly lead to the observed increase in bone strength [4, 5].

In dental medicine, the issue of implant loading as it affects the quality of implant osseointegration is still under debate. In addition to mechanical stimulation, the geometry of the implant (length and diameter) and the material's surface chemistry and topology are other parameters that positively or negatively affect osseointegration [6]. Regarding surface texture, a sandblasted, acid etched (SLA) surface is often preferred because it induces a higher bone-to-implant contact, thereby improving implant anchorage [7, 8].

The present study aimed at assessing the effect of controlled mechanical loading on the osseointegration of SLA-titanium implants inserted in rat tibiae. Loads of 1 N, 2 N, and 3 N were applied 5 days per week for 4 weeks. At the end of the stimulation protocol, the pull-out strength was taken as a primary outcome parameter of implant osseointegration. Bone microarchitecture and material properties were determined in the implants' immediate surroundings as determinant of both implant osseointegration and bone strength.

## 2. Materials and Methods

### 2.1. Experimental Design

After 2 weeks of pair feeding, two titanium implants (“test” and “anchorage”) were inserted into the right tibiae of 40 rats. The test implants were inserted to a depth of 3 mm into the secondary spongiosa of the proximal tibia, that is, without primary contact with the lower cortical shell. The anchorage implants were inserted 8 mm distally and perforated both the upper and lower cortical bone. The implants were left to heal for a period of 2 weeks after surgery to allow proper fixation in bone prior to mechanical stimulation.

The animals were then assigned to 4 groups of 10 rats, each corresponding to one level of external stimulation: nonstimulated (=NS), 1 N, 2 N, and 3 N. The duration of the stimulation protocol was 4 weeks. At 6 weeks after implantation, the animals were euthanized by an overdose of ketamine hydrochloride. The tibiae were removed for microcomputed tomographic (*μ*CT) morphometry, mechanical testing (pull-out) and nanoindentation measurements.

The experimental design and related procedures were approved by the Committee of Animal Ethics of the Faculty of Medicine, University of Geneva. The technical aspects of the activating setup were described elsewhere [9].

### 2.2. Animals and Diet

46 month-old female Sprague-Dawley rats (Charles River Laboratories, L'Arbresle, France) were acclimatized to the study conditions for a period of 2 weeks before implant placement. The animals were caged individually at 25°C. They were pair-fed a laboratory diet containing 15% casein, 0.8% phosphorus, 1% calcium, 70–80% carbohydrates, and 5% fat (Provimi Kliba AG, Kaiseraugst, Switzerland) throughout the experimental period. Demineralized water was available *ad libitum*.

### 2.3. Implant Geometry and Surface Texturing

Commercially pure grade IV titanium was selected as substrate material due to its proven compatibility with osseous tissue. Two implant geometries, that is, the “test” and the “anchorage” implant, were machined (Decobar, Yverdon, Switzerland). The test implant was 7.5 mm long and stepped in its midportion; that is, 4.5 mm was 1.4 mm in diameter and 3 mm had a diameter of 1 mm. The 4.5 mm portion was the transcutaneous part. It was surface-polished and protruded from the skin. The 3 mm portion was roughened and located within the bone. Texturing to an “SLA-like” surface was obtained by sandblasting (250 *μ*m Al_2_O_3_ at 0.5 MPa) and acid etching (10 min immersion into a mixture of 1 volume 37% HCl and 4 volumes 100% H_2_SO_4_ at 100°C). The resulting *R*_*a*_ (the mean peak-to-valley height) was 2.61 ± 0.42 *μ*m. The anchorage implant was shaped as a 8.5 × 1 mm cylinder. The polished transcutaneous part was 4 mm long. The intraosseous portion (4.5 mm) was brought to an SLA-like texture and threaded in the terminal 2 mm to allow screw fastening into the inferior cortical bone. In the protruding end of both the test and the anchorage implants, 0.3 mm vertical slits were machined to stabilize a cable that was connected to the activating device.

Prior to surgery, the implants were processed in phosphate-free cleaning solution (Deconex 15PF-x, Borer Chemie AG, Zuchwil, Switzerland), rinsed in pure water in a multifrequency ultrasonic bath, and sterilized with ethylene oxide.

### 2.4. Implantation Surgery

For implant placement, the animals were anaesthetized by abdominal injections of ketamine (100 mg/kg) and xylazine (10 mg/kg). The skin of the tibial region was shaved and cleaned bilaterally with 70% ethanol. Under aseptic conditions, the proximal medial aspect of the right tibial metaphyses was approached *via* 10 mm incisions and the surgical zones were exposed by flap reflection in medial direction. Then bores were drilled through the cortices using hand-held drills. Drilling was accompanied by profuse irrigation with saline to avoid thermal bone necrosis. The rotary speed never exceeded 2000 rpm.

The locations of the bores were set as follows. Mediolaterally, the test implant was positioned on a virtual line perpendicular to the long axis of the tibia and centrally crossing the anterior edge of the growth plate. Proximodistally the bore was placed at the intersection with a virtual line extending from the inferior border of the tendinous insertion on the proximal anterior tibial crest to the medial tendinous insertion that corresponds to the pes anserinus in humans. The test implant was manually inserted into the secondary spongiosa to a depth of 3 mm, that is, corresponding to the length of the rough portion of the implant. Accurate insertion depth was ensured by the implant's stepped design as the 1.4 mm part bottomed out against the cortical bone.

The anchorage implant was inserted on the tibia's long axis, 8 mm distal of the test implant. For these implants, both cortices were perforated using a 0.8 mm drill (corresponding to the inner diameter of the thread). The outer bore was then enlarged to 1 mm to accommodate the unthreaded portion of the implant cylinder. Eventually, the anchorage implant was manually screw-tightened into the apical cortical shell.

After insertion of the implants, the skin flap covering the implants was sutured back into position using 3–0 resorbable polylactic suture (Vycril; Ethicon, Spreitenbach, Switzerland). Postoperative pain control was achieved by subcutaneous injection of buprenorphine (0.06 mg/kg) twice a day for 3 days. Those implants that did not spontaneously perforate the skin were exposed surgically after two weeks.

### 2.5. Implant Loading

**Figure 1:**
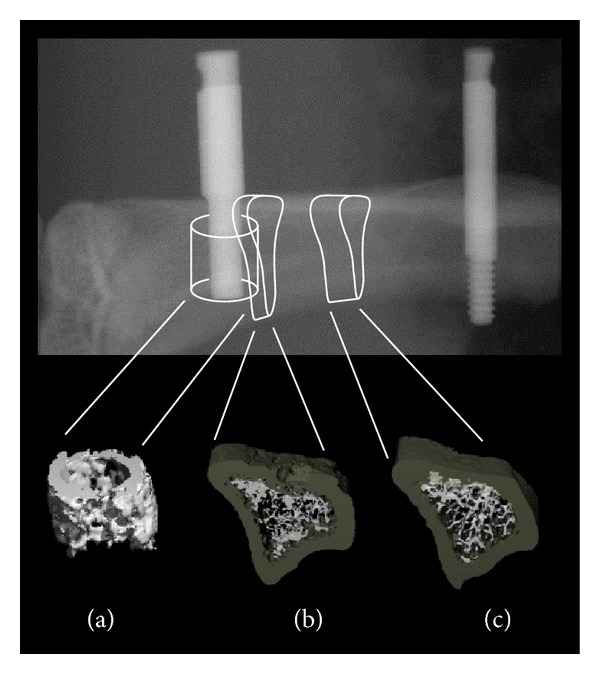
Radiograph of the implants inserted into the proximal tibial metaphysis. Diagrammatic view showing the position of the test (left) and the anchorage (right) implants. The 3 regions of interest (ROI) are shown. (a) Peri-implant band of bone located around the implant's most apical segment that was still exclusively surrounded by trabecular bone. Height: 56 slices (ca. 1.12 mm), band width: 0.5 mm. (b) ROI number two. Volume of 50 slices (ca. 0.98 mm) including both the cortical and trabecular compartment of bone. Slice number 1 was located immediately outside the distal surface of the test implant. (c) ROI number three. Volume of 100 slices (ca. 1.98 mm) contoured midway between the two implants and also including both the cortical and the trabecular compartments of bone.

At the onset of the experiment, we aimed at generating peak intra-osseous strains in the 500 to 2500 *με* range. To set the appropriate external load levels, 5 rat tibiae fitted with two implants were converted to finite element meshes [10] and provided with pertinent density dependent material properties [11]. After numerical simulation of the experimental loading conditions, forces of 1, 2 and 3 N were found to correspond to average strains of 700 ± 200 *με*, 1400 ± 400 *με*, and 2100 ± 600 *με* in the bone corresponding to ROI-b in Figure 1. Validation of the simulation using strain gauges on *ex-vivo* specimens yields errors in 15% range.

Therefore, four groups were established, that is, one control (nonstimulated, NS) and three groups activated to peaks forces of 1, 2, and 3 N.

During the loading procedure, all rats (i.e., including the NS group) were laying on their back and sedated with isofluorane oxygen through a snout mask. The loading machines [9] were driven by rotary voice coil actuators (BEI, Kimco Magnetics) that generated sinusoidal pulse-release movements. The actuators were connected to the implant heads by cables fitting into the slits. The geometry of the setup thus forced both implant heads together and generated a pattern of tensile and compressive stress fields inside the surrounding bone bed [9].

The implants were stimulated five days per week for 900 sinusoidal half-cycles at a frequency of 1 Hz. At the inception of the activation period, the load levels were augmented gradually by daily increments of 0.5 N until the prescribed level was reached.

### 2.6. Microcomputed Tomographic (*μ*CT) Morphometry

Immediately after death, the animals' tibiae were dissected at the joints and the soft tissues were removed. They were individually wrapped in saline-soaked gauze and frozen to −20°C in plastic bags. 12 hours prior to *μ*CT analysis, the bones were left to thaw to 4°C. They were then brought to room temperature and sectioned ca. 1 cm distal to the anchorage implant. For scanning, they were placed in 2 cm diameter tubes filled with saline solution.

Microarchitecture and bone-to-implant contact were evaluated by *μ*CT morphometry using a high-resolution *μ*CT system (*μ*CT 40; Scanco Medical, Bruettisellen, Switzerland). The system's voxel size was 20 *μ*m in all spatial directions. For penetrating radioopaque titanium and optimizing the signal-to-noise ratio, the system was set to 70 kEV and 350 ms integration time. The number of tomographic projections was 1000 per 360°. Images were filtered with a 3D Gauss filter, and bone and titanium were segmented individually using two distinct thresholds values, which were visually chosen. For bone, the segmentation parameters were set to sigma: 0.7 voxels, support: 1 and threshold: 2,5 cm^−1^, for titanium, sigma: 1.5 voxels, support: 2, and threshold: 7,6 cm^−1^.

The bone's microstructure was analyzed in three discrete regions of interest (ROIs) (Figure 1). The first ROI was located along the long axis of the test implant in the most apical segment that was still exclusively surrounded by trabecular bone. The height of this ROI was 56 slices (ca. 1.12 mm) and encompassed a peri-implant circular band of trabecular bone, 0.5 mm (Figure 1(a)). Relative bone volume (BV/TV), trabecular number (Tb.N), trabecular thickness (Tb.Th), and trabecular spacing (Tb.Sp) were computed from the 3D distances within the trabecular network. The structure model index (SMI) (i.e., an estimate of the plate-rod characteristics of trabecular bone) was also calculated (for an ideal plate structure SMI = 0; for an ideal rod structure SMI = 3). The two other ROIs were located (i) in the immediate vicinity of the test implant and (ii) between the two implants. More specifically, ROI number two (Figure 1(b)) encompassed a volume of 50 slices starting from the distal edge of the implant. Slice number 1 was located immediately outside the test implant and the remaining 49 were contoured by moving in distal direction toward the anchorage implant. This ROI measured ca. 0.98 mm in length and included both the cortical and trabecular compartment of bone. ROI number three (Figure 1(c)) was a volume of 100 slices contoured midway between the two implants. For its positioning, the edges of the test and the anchorage implants were taken as landmarks and the middistance was determined. Then 50 slices were generated on both sides, that is, moving in both directions along the proximodistal axis of the tibia. This ROI measured ca. 1.98 mm in length and also included both the cortical and the trabecular compartments of bone.

The gray-scale images were segmented with a 3D Gauss filter so that the trabecular and cortical portions could be segregated by thresholding. In ROIs number two and three, the mean cortical thickness was determined.

### 2.7. Pull-Out Test

After *μ*CT analysis, the implants were subjected to pull-out testing. To this end, the tibiae were held with a metal jig while a metal clasp was affixed to the test implants' head and was used to grip the implant during pulling. Pull-out strength was determined as the peak force (i.e., the maximum failure force) applied when detaching the implant from the bone. The test was conducted using a servo-controlled electromechanical system (Instron 1114; Instron Corp., High Wycombe, UK) with the actuator displacement set to 2 mm/min. A preliminary study had demonstrated that freezing the tibiae did not notably alter pull-out strength values [1] as the regression between values generated before and after freezing was *r* ^2^ = 0.96.

### 2.8. Nanoindentation

The material level properties of the bone tissue were characterized by nanoindentation [12]. The measurements were performed with a nanohardness tester (NHT, CSM Instruments, Peseux Switzerland). The test consisted in driving a pyramidal diamond indenter into the bone surface thereby generating a force-displacement curve. The curves thus obtained are combinations of plastic, elastic, and postyield deformation which are then numerically processed to obtain the E-modulus, the tissue's hardness, and the working energy developed during indentation (W). The principles and procedures related to nanoindentation measurements are detailed in Hengsberger et al. [13].

Technically, after the pull-out tests were completed, the tibiae were embedded in polymethylmethacrylate (PMMA) resin and the blocks were transversally sectioned into two pieces with a diamond wire saw midway between the test and the anchorage implant. The surface facing the test implant was polished and finished with 0.25 *μ*m diamond paste and the specimens were stored at −20°C until further processing. In the night before testing, they were thawed to 4°C in saline solution and then gently brought to room temperature. Nanoindentation consisted in placing 10 homogenously distributed indents into the trabecular bone of the polished surface. The indents were brought to a depth of 900 nm at a loading rate of *F*/*t* = 76 mN/min, both during loading and unloading. The indenter was maintained at maximal depth for 5 s. The applied load and the penetration depth were continuously recorded during the loading and unloading cycle. The tissue's hardness (H) and E-modulus (E) were calculated from the load-displacement curve as described by Oliver and Pharr [14]. The working energy was taken as the surface under the curve. Specimens were kept in saline solution before and after testing.

### 2.9. Statistical Analysis

All results were expressed as means ± SEM. For normally distributed data, significant differences were identified by analyses of variance (ANOVA) and Fisher's post hoc test. Alternatively Mann-Whitney *U* tests were performed. The level of significance was set to *P* ≤ 0.05.

## 3. Results

### 3.1. Effect of Mechanical Loading on Pull-Out Strength

**Figure 2:**
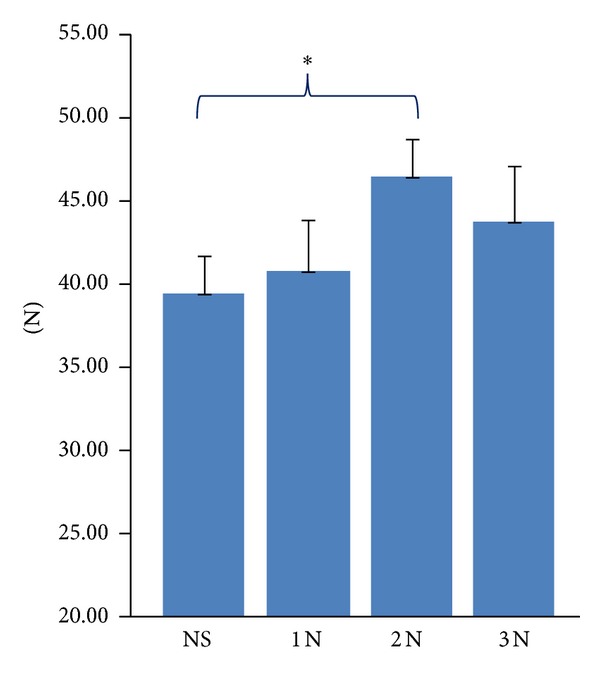
Effect of 4 weeks of *in vivo* mechanical loading on pull-out strength. The pull-out strength measured in the 2 N group was significantly higher compared to NS. *P < 0.05. Means ± SEM. NS: nonstimulated.

4 weeks of stimulation increased the pull-out strength of the test implants in all groups (Figure 2); higher values were observed in the 2 N group (+17.8% *P* < 0.05) compared to the NS and the 3 N group (+10.7%).

### 3.2. Effect of Mechanical Loading on the Trabecular Microarchitecture of Bone Surrounding the Implant

Compared to the NS group, mechanical loading did not significantly alter the parameters of bone microarchitecture within 0.5 mm of the test implant (Figure 1(a)). Nonetheless tendencies were noted. BV/TV moderately increased in all groups compared to NS and higher values were obtained at 1 N (+14%), while the 2 N and 3 N groups presented augmentations of less than 10% (Table 1). A similar trend was observed for trabecular thickness and trabecular number (resp., +8% and +5% in the 1 N group versus NS). These changes, however, were not significant.

### 3.3. Effect of Mechanical Loading on Trabecular Bone Microarchitecture and Cortical Thickness of Bone in Selected Zones in the Vicinity of the Test Implant

Further parameters of bone microarchitecture were determined in ROI number two located distally to the test implant (Figure 1(b)). The ROI was 50 slices in depth (ca. 0.98 mm) and included both the cortical and the trabecular compartment of the bone. In this zone, BV/TV was significantly higher in the 1 N group than in the 3 N group (+46% *P* < 0.05) and increased by 38% compared to NS (Table 2). Similarly, trabecular thickness was 13% higher in the 1 N versus the 3 N group (*P* < 0.06) and +8% compared to NS. A minor change was observed in trabecular number (+16% in the 1 N versus the 3 N group and +14% versus NS). The cortical bone's thickness significantly increased in the 3 N group as compared to 1 N (+7% *P* < 0.05) but did not notably change when compared to NS.

### 3.4. Effect of Mechanical Loading on Trabecular Bone Microarchitecture and Cortical Thickness of Bone Located Midway between Both Implants

Bone microarchitecture was also assessed in ROI number three (100 slices, ca. 1.98 mm) located midway between both implants (Figure 1(c)). The 1 N group displayed significantly higher BV/TV (+89% *P* < 0.01) compared to the 3 N group (Table 3). The trabecular number was significantly higher for 1 N compared to 3 N (+46% *P* < 0.01), while the trabecular thickness did not appreciably change. The structure model index was 15% higher in the 3 N-compared to 1 N group indicating a more rod-like structure (versus plates) of the trabecular units. Cortical thickness slightly increased in the 3 N versus NS, 1 N, and 2 N groups, although these changes were not statistically significant.

### 3.5. Effect of Mechanical Loading on the Intrinsic Quality of Trabecular Bone Midway between Both Implants

Mechanical stimulation significantly increased the modulus of elasticity of the trabecular bone in the 2 N compared to all other groups (+15% versus NS; +12% versus 1 N; +13% versus 3 N) (Table 4). Hardness was significantly higher in the 2 N group versus NS (+9% *P* < 0.05) and 3 N group (+14% *P* < 0.05). The working energy was higher in trabecular bone in all stimulated groups, reaching significance in the 1 N and 3 N groups versus NS (+15%). For all measurements, the coefficients of variation of E, H, and W ranged from 3.5 to 6%.

The effects are summarized in Table 5.

## 4. Discussion

As a first goal of the present study, we investigated the effect of *in vivo* mechanical microstimulation on implant osseointegration, using a rat model. Three different levels of stimulation (1 N, 2 N, and 3 N, that is, strains of 700 ± 200 *με*, 1400 ± 400 *με*, and 2100 ± 600 *με*) were applied and the dose-response on bone microarchitecture and material level properties were investigated. The pull-out force of the implant from the bone bed was selected as primary outcome as it denoted the combined effect of the determinants of osseointegration, that is, the trabecular and cortical bone microarchitecture and the geometry and texture of the implant surface (which was maintained constant during fabrication procedures). A second goal was to assess the effect of load magnitude on bone microarchitecture and bone material level properties in a ROI taken midway between the two implants.

As expected, there were definite biological variations in the response induced by the various load levels applied. In this regard, a number of parameters might account for the variability observed; that is, (i) the healing process, as the animals reacted differently to implant placement—some developing erythematous inflammatory reactions around the protruding implant; (ii) in spite of the implants maintaining their osseointegration, changes in the mechanical environment during the 4-week stimulation period were likely. Indeed, although the stepped implant accurately set the length of the lever at placement, individual variations of peri-implant bone mass due to loading affected the moment applied to the bone beds and thus altered the strain distribution around the implants. An estimate of the variations thus induced is in the 10% range.

A self-evident approach to alleviate the effects of biological variability is to augment the number of units in each group. Problematically, the microstimulation procedure is labor intensive and, in the present setting, only 32 animals could be processed each day—the reason for maintaining the number of animals at 10 in each group. Still, in spite of biovariability, the present data demonstrate a significant effect of mechanical stimulation on pull-out resistance.

Two freezing-thawing cycles were included into the testing workflow and these cycles might have altered the bone's mechanical properties. Several mechanisms were postulated to this effect, that is, either by freezing expansion of the water or by damage to the collagenous matrix. Experimental data range from “no effect” [15] to “slight damage” (maximum 15%) [16]. In a recent study, the freezing of rat tibiae was found to have no effect on the bone-implant interface [17]. Also, if an effect exists, all reports concur to state that mechanical properties are degraded. Consequently, in our experiment, all groups were affected to the same degree and intergroup comparisons remained valid.

The applied stimulation at 1 Hz is an adequate frequency to induce a reaction to loading [18, 19]. The pull-out strength was significantly augmented in animals stimulated at a force level of 2 N as compared to the NS group; this positive effect was also observed in the 3 N group but did not reach the level of significance. Regarding forces in excess of 3 N (i.e., 2100 ± 600 *με*), their effect on implant osseointegration is not known and requires further investigations.

In previous studies, we clearly established the role played by the microarchitecture, bone-implant contact, bone material level properties, and surface of the implant as the determinants which mostly affected the bone bed's resistance to pull-out [1–3, 20]. In the present study, the pull-out resistance was maximized under 2 N when compared to NS rats; therefore we performed a systematic evaluation of the potential determinants of pull-out strength in order to better understand the mechanism involved. Using *μ*CT analysis, we first investigated whether mechanical loading induced any changes in bone microarchitecture in a cylindrical volume of trabecular bone taken 0.5 mm around the test implants (Figure 1, ROI-a). However, in this ROI, no notable changes in trabecular morphology (as expressed by its BV/TV) were evidenced. Hence BV/TV provided no explanation as to the augmented implant anchorage under 2 N loading. We hypothesize that the effect on bone formation in the vicinity of the implant may be due to a further ingrowth of bone into the surface irregularities of the SLA implant and hence an improvement in osseointegration [21–23]. Consequently the differences observed in pull-out force derive from a stronger mechanical interlock of the bone forming at the interface with the microrough titanium in the 2 N group. Unfortunately *μ*CT analyses do present technical limitations in this regard as their resolution does not permit to adequately quantify the bone growing within the interstices of the titanium's acid etched surface. This issue might be investigated further by analyzing the characteristics of the bone-implant interface using back-scattered electron microscopy. In addition a mapping of this subregion using synchrotron radiation would allow the accurate 3D assessment of the bone tissue penetrating into the crevices of the implant surface [24–26]. In view of the present data, efforts to access this technology are planned. Histology and histomorphometric quantifications of cellular activity at trabecular and endosteal/periosteal surfaces are warranted as complementary strategies to assess the effect of loading on modeling/remodeling of bone adjacent to the implant.

The second goal of this study was to investigate the effect of the mechanical stimulus in two other segments of the tibia in the vicinity of the test implant. To this end, we analyzed two additional ROIs, that is, cortical and trabecular bone immediately distal to the test implant (Figure 1(b)) and midway between both implants (Figure 1(c)). In these two ROIs, two trends regarding trabecular and cortical bone emerged. Trabecular bone reacted favorably to lower levels of mechanical loading, that is, 1 N (i.e., 700 ± 200 *με*). Conversely, notable deleterious effects were observed for stimulations in the 3 N range (i.e., 2100 ± 600 *με*). According to Frost's concept of functional adaptation to mechanical stimulation [27], bone mass increases when the “minimum effective strains” rise above a definite threshold. In a situation of “mild overloading” the activation of basic multicellular units (BMUs) results in net bone formation (similar to that observed in trabecular bone in the 1 N group (Figure 1, ROI-b and ROI-c)).

Tibia is a load bearing bone. Therefore we hypothesize that strains due to the animals' daily activities combine with the strains generated by the actuators on the implants. Under this premise, forces of low magnitude (1 N) may result in an osteogenic stimulus thus explaining the increase in trabecular BV/TV observed in the 1 N versus the 3 N group. In contrast, bone “overloading” would induce rapid regional bone activation and remodeling but also increase the density of microdamages. In the latter instance, the BMUs' capability for repair might be exceeded thus resulting in net bone loss. We hypothesize that, when loaded to 3 N, a blend of anabolic and resorptive phenomena is established. Whether cortical and trabecular bone share the same threshold is a matter of debate. Still, by virtue of its sheer mass, cortical bone would be loaded to a “mild overload” and increase in mass while the trabecular network would be “overloaded” and resorb.

Cortical bone thickness was also affected by external mechanical loading. A significant increase was observed when a force of 3 N was applied—1 and 2 N being ineffective. Interestingly this effect was restricted to bone in close vicinity of the implant (Figure 1(b)) and was not demonstrated in the bone located midway between both implants (Figure 1(c)). This anabolic effect of mechanical loading on the cortex is in agreement with another model in which the application of external loading on mice/rat tibiae or radii was shown to induce periosteal apposition and thickening of the cortex [4, 28–32]. In a similar way to with trabecular bone, dynamic parameters of bone formation using periosteal and endosteal histomorphometry at increasing distance from the test implant would permit a quantification of the load-induced response of the cortices along the long axes of the tibiae.

Finally, we know that the bone's material properties positively affect implant osseointegration. This was demonstrated in animals treated with strontium ranelate—a medication which improved both intrinsic bone tissue quality and pull-out strength (taken as an outcome parameter of osseointegration) [2]. In the present study bone material level properties were measured in transversal sections cut midway between the two implants and after the pull-out test. The modulus of elasticity and hardness significantly increased in the 2 N group as compared to the NS and the 3 N groups. The same holds for working energy which significantly increased in the 1 N and 3 N groups compared to NS. These findings account for a load-dependent change in bone material level properties within the midimplant ROI (Table 4). Tissues in this segment thus are affected by the loading of the test implant. Conversely, the analysis of bone tissue close to the test implant is problematic. One reason is the interference of shear stresses due to the presence of titanium which may corrupt the assessment of the bone tissue's mechanical properties by nanoindentation. Also, in the present study design, the pull-out test affected the integrity of the bone in the immediate vicinity of the implant, thus precluding any form of nanoindentation test. Still, measurements taken midway between the two implants indicate a global effect of implant loading on the bone tissue's properties in the 2 N group and a similar effect at the bone-implant interface is likely. Indeed a positive effect of implant microstimulation on material level properties at the bone-implant interface could add up to the change in bone microstructure within the acid etched SLA surface and would explain the increased pull-out strength observed in the 2 N group. In a previous study in which different regions of the rat vertebral body were systematically investigated, we demonstrated that the zones subjected to the largest loads also presented the highest values of material level properties [13]. The mechanism, though, of this interaction is not fully elucidated. Still, when mice selectively overexpress IGF-I in osteoblasts, the deleterious effects of a low-protein diet on bone material level properties are minimized. This observation might indicate that IGF-I production in bone could favorably affect bone material level properties [33]. Since IGF-I mRNA is stimulated by mechanical loading [34–36] such a pathway could be involved.

## 5. Conclusions

A broad view at the present findings on the interplay between load effects and bone determinants indicates that stimulation at 2 N (=1400 ± 400 *με*) maximized the pull-out strength.

The *in vivo* mechanical loading of endosseous implants induces load-dependent modifications in bone microarchitecture. Depending on the load level, the effects primarily affect the trabecular or the cortical compartment of the bone or the material level properties. Further investigations will refine our understanding of each determinant as it responds to the external load applied.

## Figures and Tables

**Table 1:** Effect of 4 weeks of mechanical loading on bone microarchitecture measured in a 0.5 mm circular band selected around the implant test in rat tibiae.

	NS	1 N	2 N	3 N
BV/TV%	44.2 ± 3.1	50.5 ± 2.3	48.5 ± 1.9	48.0 ± 3.9
Tb.N (1/mm)	6.23 ± 0.177	6.53 ± 0.200	6.02 ± 0.234	6.27 ± 0.294
Tb.Th (mm)	0.142 ± 0.003	0.153 ± 0.006	0.145 ± 0.003	0.149 ± 0.005
Tb.Sp (mm^3^)	0.169 ± 0.008	0.160 ± 0.012	0.174 ± 0.010	0.168 ± 0.015
SMI	−0.155 ± 0.316	−0.595 ± 0.344	−0.487 ± 0.222	−0.388 ± 0.370
Contact (%)	76.02 ± 2.66	78.73 ± 2.25	78.35 ± 2.19	76.85 ± 3.70
POS (N)	39.57 ± 2.23	40.82 ± 3.12	46.63 ± 2.21*	43.81 ± 3.41

Mean ± SEM.

*P < 0.05 compared to NS.

POS: pull-out strength.

**Table 2:** Effect of 4 weeks mechanical loading on parameters of bone microarchitecture analyzed in a region of 50 lines selected near the implant test in rat tibiae.

	NS	1 N	2 N	3 N
BV/TV%	14.6 ± 2.2	20.1 ± 2.2^#^	16.9 ± 2.1	13.8 ± 2.2
Tb.N (1/mm)	3.74 ± 0.27	4.26 ± 0.29	4.03 ± 0.23	3.67 ± 0.14
Tb.Th (mm)	0.074 ± 0.002	0.080 ± 0.003	0.074 ± 0.003	0.071 ± 0.004
Tb.Sp (mm^3^)	0.28 ± 0.03	0.24 ± 0.02	0.25 ± 0.02	0.28 ± 0.01
SMI	2.7 ± 0.15	2.3 ± 0.15	2.5 ± 0.14	2.7 ± 0.18
Ct.Th (mm)	0.44 ± 0.01	0.43 ± 0.01	0.43 ± 0.01	0.46 ± 0.01*

Mean ± SEM.

^#^ *P* < 0.05 versus 3 N; *P < 0.05 versus 1 N.

**Table 3:** Effect of 4 weeks of mechanical loading on parameters of bone microarchitecture analyzed in a region of 100 lines selected between the test and the anchorage implants in rat tibiae.

	NS	1 N	2 N	3 N
BV/TV%	8.3 ± 1.4	11.5 ± 1.2^#^	8.2 ± 1.3	6.1 ± 1.1
Tb.N (1/mm)	2.77 ± 0.22	3.20 ± 0.22^#^	2.58 ± 0.29	2.19 ± 0.17
Tb.Th (mm)	0.071 ± 0.002	0.071 ± 0.002	0.071 ± 0.002	0.071 ± 0.005
Tb.Sp (mm^3^)	0.38 ± 0.03	0.33 ± 0.03	0.47 ± 0.12	0.48 ± 0.03
SMI	2.99 ± 0.10	2.78 ± 0.10	3.00 ± 0.15	3.19 ± 0.14°
Ct.Th (mm)	0.49 ± 0.02	0.48 ± 0.02	0.48 ± 0.03	0.51 ± 0.02

Mean ± SEM.

^#^ *P* < 0.01 versus 3 N; °*P* < 0.05 versus 1 N.

**Table 4:** Effect of 4 weeks of mechanical loading on intrinsic quality of bone analyzed between the test and the anchorage implants in rat tibiae.

	NS	1 N	2 N	3 N
E (GPa)	14.2 ± 0.5	14.5 ± 0.4	16.3 ± 0.4^∗∗a,b^	14.4 ± 0.4
H (MPa)	552 ± 20	578 ± 17	604 ± 17^∗b^	528 ± 14
W (pJ)	3491 ± 115	4014 ± 97**	3785 ± 135	4024 ± 92**

Mean ± SEM.

*P < 0.05 NS; **P < 0.001 versus NS.

^a^ *P* < 0.01 versus 1 N; ^b^ *P* < 0.01 versus 3 N.

E: modulus of elasticity; H: hardness; W: working energy.

**Table 5:** The sum of the effects of 4 weeks of mechanical loading on the determinants of pull-out strength in rat tibiae.

	1 N	2 N	3 N
Trabecular bone	+	0	−
Cortical bone	0	0	+
Intrinsic bone quality	±	+	±
Pull-out strength	0	+	±

## Abbreviations

NS: Nonstimulated
1 N-2 N-3 N: Value of the applied force in Newton
BV/TV: Bone volume to total volume
Tb.N: Trabecular number
Tb.Th: Trabecular thickness
Tb.Sp: Trabecular space
SMI: Structure model index
Ct.Th: Cortical thickness
E: Modulus of elasticity
H: Hardness
W: Working energy.
